# Metal-Promoted Assembly of Two Collagen Mimetic Peptides into a Biofunctional “Spiraled Horn” Scaffold

**DOI:** 10.3390/ma9100838

**Published:** 2016-10-17

**Authors:** Kevin Strauss, Jean Chmielewski

**Affiliations:** Department of Chemistry, Purdue University, 560 Oval Drive, West Lafayette, IN 47907, USA; kstrauss@purdue.edu

**Keywords:** collagen mimetic peptides, hierarchical assembly, biomimetic scaffold

## Abstract

Biofunctional scaffolds for the delivery of living cells are of the utmost importance for regenerative medicine. Herein, a novel, robust “spiraled horn” scaffold was elucidated through the Co^2+^-promoted hierarchical assembly of two collagen mimetic peptides, **NCoH** and **HisCol**. Each “horn” displayed a periodic banding pattern with band lengths corresponding to the length of the collagen peptide triple helix. Strand exchange between the two peptide trimers resulted in failure to form this intricate morphology, lending support to a precise metal-ligand-based mechanism of assembly. Little change occurred to the observed morphology when the Co^2+^ concentration was varied from 0.5 to 4.0 mM, and the scaffold was found to be fully formed within two minutes of exposure to the metal ion. The horned network also displayed biological functionality by binding to a His-tagged fluorophore and associating with cells.

## 1. Introduction

Collagen is a crucial component of the extracellular matrix, supporting the growth and development of cells [1]. It is a robust biopolymer that adopts a highly stable triple helix morphology [2,3]. As such, collagen is a versatile biomaterial, having previously been assembled into bioadhesive microparticles [4,5], largely through emulsification [6] and microfluidic [7,8] techniques. However, a more practical and cost-effective alternative is the use of collagen mimetic peptides (CMPs), designed with specific sequences to promote self-assembly through native chemical ligation [9], cationic-π interactions [10], cysteine bridge formation [11,12], electrostatic interactions [13,14], hydrophobic packing [15,16], and metal-ligand interactions [17], the latter of which is described in more detail below. Recent examples of other such bioinspired approaches include the use of a short collagen peptide in which proline residues were replaced with non-canonical (2S, 4R)-4-aminoproline to electrostatically direct self-assembly into nanosheets [14], as well as conjugation of a thermoresponsive polymer to a different CMP to thermally direct its reversible self-assembly into nanovesicles [18]. Still other studies made use of a “sticky-ended” nucleation strategy, inspired by the assembly of DNA and coiled-coiled helices, in which staggered collagen helices, generated through the exploitation of lysine-aspartate axial salt bridges, were pieced together into microfibers [19]. However, few strategies have proven to be as elegant and as versatile as metal-promoted self-assembly. 

**Figure 1:**
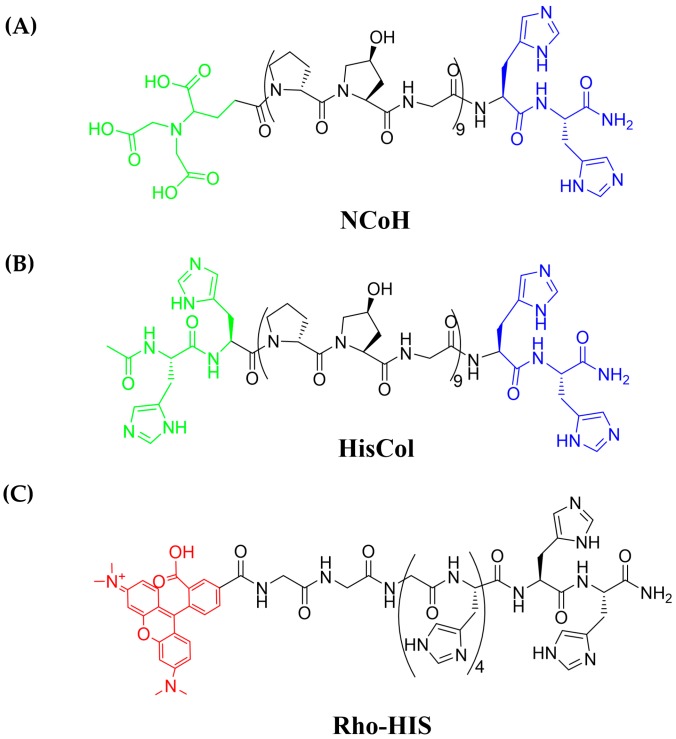
Sequences of (**A**) **NCoH**; (**B**) **HisCol**; and (**C**) **Rho-HIS**.

**Figure 2:**
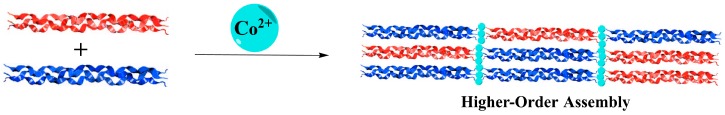
Proposed mechanism of assembly of **NCoH** (**red**) and **HisCol** (**blue**) in the presence of CoCl_2_ in aqueous buffer (MOPS, pH 7.1).

Metal-promoted self-assembly has been investigated by taking advantage of precisely-positioned ligands on the triple helical backbone of collagen mimetic peptides in order to form a variety of three-dimensional morphologies after incubation with metal ions. Numerous morphological variations resulted from the placement and type of metal-binding ligands included in the sequence and the species of metal used. For instance, the radial placement of bipyridine ligands along the central portion of the triple helix resulted in the metal-promoted formation of fibers [20], disks [21], and hollow microspheres [22], the latter capable of the encapsulation and thermally-controlled release of bioactive cargo [23]. A linear strategy with placement of ligands at the termini of collagen peptides resulted in metal ion-assisted assembly into micron-sized florettes and an extended petal-like structure [24,25]. A peptide with sequence (Histidine)_2_–(proline-hydroxyproline-glycine)_4_-(proline-hydroxyproline(Bipyridine)-glycine)-(proline-hydroxyproline-glycine)_4_-(Nitrilotriacetic acid), otherwise known as **HBN**, was designed via a cross-linked approach, in which metal-binding ligands were positioned at both the central region and the termini of the triple helix. In the presence of Ni^2+^, **HBN** assembled into a three-dimensional matrix, capable of encapsulating HeLa cells [26] and MCF10A cells, and supporting the formation of MCF10A spheroids [27]. The present work centers on two collagen mimetic peptides with the linear design motif. The first peptide, (Nitrilotriacetic acid)-(proline-hydroxyproline-glycine)_9_-(Histidine)_2_, also known as **NCoH**, contains a nitrilotriacetic acid (NTA) ligand at the *N*-terminus of the (proline-hydroxyproline-glycine)_9_ ((POG)_9_) backbone and a dihistidine (di-His) moiety at the C-terminus (Figure 1A). Earlier work demonstrated that, in the presence of a variety of divalent metal ions, including Co^2+^, **NCoH** undergoes self-assembly into microflorettes, ruffled spheres that measure 10–20 μm in diameter [24]. Two analogs of **NCoH** were also generated: (His)_2_-(POG)_9_-(His)_2_, heretofore referred to as **HisCol**, which contains the dihistidine motif at both termini (Figure 1B), and (Iminodiacetic acid)-(POG)_9_-(Iminodiacetic acid), otherwise known as **IdaCol**, which contains the iminodiacetic acid (Ida) moiety at each terminus. A 1:1 mixture of **HisCol**/**IdaCol** was found to assemble into a petal-like assembly in the presence of Ni(II) [25]. **NCoH** forms higher-order assemblies through aggregation of triple helical units in a head-to-tail fashion, driven by the formation of stable NTA-M^2+^-(His)_2_ complexes at the termini [24]. Assembly of **HisCol**/**IdaCol** complexes has been proposed to occur in a tandem fashion, with Ida-M^2+^-(His)_2_ complexes between the alternating units [25]. Due to the striking structural similarities between **NCoH** and **IdaCol**, we propose that substitution of **NCoH** for **IdaCol** with **HisCol** should result in new possibilities for metal-promoted assembly of collagen mimetic peptides (Figure 2). The characterization of the resulting assembly is presented herein.

## 2. Materials and Methods

### 2.1. Materials

The Rink Amide ChemMatrix resin, used in solid phase peptide synthesis, was commercially obtained from Pcas-Biomatrix Inc. (Quebec, QC, Canada). Fmoc-protected amino acids and *O*-benzotriazole-*N*,*N*,*N′*,*N′*-tetramethyluraniumhexafluorophosphate (HBTU) were purchased from AAPPTec (Louisville, KY, USA), ChemPep, Inc. (Wellington, FL, USA), and Chem-Impex International (Wood Dale, IL, USA). Piperidine and diisopropylethylamine (DIEA) were procured from Alfa Aesar (Ward Hill, MA, USA). Acetonitrile, triisopropylsilane (TIPS), trifluoroacetic acid (TFA), and cobalt (II) chloride hexahydrate were all obtained from Sigma Aldrich (St. Louis, MO, USA). *N*,*N*-dimethylformamide (DMF), dichloromethane (DCM), and methanol (MeOH) were purchased from AVANTOR (Center Valley, PA, USA) and Fisher Scientific (Pittsburgh, PA, USA). Ethylenediaminetetraacetic acid disodium salt (EDTA) was purchased from Mallinckrodt JT Baker (Hazelwood, MO, USA). Scanning electron microscopy (SEM) stubs, round glass coverslips, and transmission electron microscopy (TEM) grids were obtained from Ted Pella, Inc. (Redding, CA, USA).

### 2.2. Synthesis of Peptides

Standard Fmoc-based solid-phase peptide synthesis techniques were used to generate the peptides **NCoH** and **HisCol** starting from H-Rink amide resin and using six equivalents of the desired amino acid, six equivalents of HBTU, and 12 equivalents of DIEA for each coupling. The resin was washed with DMF, DCM, MeOH, again with DCM, and again with DMF. The Fmoc-protecting group was removed using 25% piperidine in DMF (v/v). Couplings were monitored using the Kaiser [28] or the chloranil tests [29]. This process was continued until the sequence (POG)_9_-HH-NH-resin was synthesized. For **HisCol**, two additional histidine residues were then coupled to the *N*-terminus to form HH-(POG)_9_-HH-NH-resin. Upon completion of the final coupling and subsequent washing, the peptide was capped using 5% acetic anhydride and 8.5% DIEA in DMF with agitation for one hour. For **NCoH**, the final acylation of the free amine occurred with NTA (4 eq.) in the presence of HBTU (4 eq.) and DIEA (8 eq.). For the His-tagged peptide fluorophore rhodamine-(Gly)_3_-(His)_6_, known as **Rho-HIS** (Figure 1C), solid-phase peptide synthesis proceeded as described above until the sequence (Gly)_3_-(His)_6_-NH-resin was formed. The final acylation was accomplished by loading the flask with NHS-rhodamine (1.5 eq.), HBTU (6 eq.), and DIEA (12 eq.) in DMF and agitating in the dark for 3 h. The solution was drained, and the resin was washed as before. Coupling was verified with the Kaiser test. A TFA cleavage cocktail (95% TFA, 2.5% TIPS, 2.5% H_2_O, 15 mL) was added to each of the resin-bound peptides. The resin was agitated at room temperature for three hours, the solvent was removed in vacuo, and the peptide was precipitated with cold diethyl ether (50 mL × 2 × 30 min). The solution was centrifuged at high speed for 10 min, and the solvent was removed by decanting. This process was repeated once. The crude material was characterized by matrix-assisted laser desorption ionization—time of flight mass spectrometry (MALDI-TOF MS) and analytical high-pressure liquid chromatography (HPLC). The desired peptides were purified to homogeneity by HPLC on a semi-prep Luna C18 column (Phenomenex, Torrance, CA, USA). The pure peptide fractions were collected, and the solvent was removed in vacuo to afford pure **NCoH**, **HisCol**, and **Rho-HIS**, which were characterized by MALDI-TOF mass spectrometry: **NCoH**: calculated: 2938.02 Da, observed: 2936.02 Da; **HisCol**: calculated: 3013.20 Da, observed: 3013.55 Da; **Rho-His**: calculated: 1424.49 Da, observed 1424.76 Da.

### 2.3. Metal-Ion-Promoted Assembly

To a 1.5 mL Eppendorf tube was added 3-(*N*-morpholino)propanesulfonic acid (MOPS) buffer, pH 7.1 (20 mM, 10 μL), DI H_2_O (20 μL), **NCoH** and **HisCol** peptides (1.0 mM each in H_2_O, 10 μL), and CoCl_2_ (2.0 mM, 10 μL). The solution was vigorously mixed, and the Eppendorf tube was sealed and allowed to incubate overnight (~16 h). The visible precipitate was centrifuged at 10,000× *g* for three minutes at 4 °C, and 45 μL of the supernatant was removed. The solid residue was resuspended in H_2_O (45 μL), and this process was repeated two more times. The resulting residue was resuspended in H_2_O (45 μL) and prepared for imaging.

### 2.4. Scanning Electron Microscopy (SEM)

Metal-peptide assemblies were prepared in 50 μL volume as described above. For each sample to be examined, a round glass coverslip was affixed to the surface of an SEM stub by means of double-sided copper tape. A 10 μL aliquot of each sample was plated onto separate stubs. Samples on stubs were then freeze-dried to prevent flattening of the sample. Immediately prior to imaging, samples were sputter-coated with platinum for 60 s. Samples were visualized using a FEI Nova nanoSEM field emission SEM (FEI Company, Hillsboro, OR, USA) equipped with an Everhart-Thornley detector (ETD) for lower resolution scans and a through-the-lens (TLD) detector for higher resolution, immersion imaging. For all experiments, the instrument was operated at an accelerating voltage of 5 kV with a working distance of 5 mm, a spot size of three, and a 30 μm aperture.

### 2.5. Transmission Electron Microscopy (TEM)

Metal-peptide assemblies were prepared in 50 μL volume as described above. Prior to sample mounting, 400-mesh copper grids coated in Formvar with a carbon film (Ted Pella, Inc., Redding, CA, USA) were glow discharged. A 3 μL aliquot of sample was placed directly on the grid. The grid was allowed to sit with the droplet for three minutes. The liquid was then wicked away with filter paper. The grid was treated with 2% uranyl acetate (pH ~4) by using forceps to pass the grid through a droplet of the stain. Excess stain was then wicked away with filter paper. Grids were allowed to air dry for one minute. This process was repeated for every sample to be examined. Samples were visualized using a Tecnai T20 (FEI Company, Hillsboro, OR, USA) at an accelerating voltage of 200 kV, a spot size of one, and with a 200 μm condenser aperture and a 70 μm objective aperture in place. Images were obtained with a SIA L3C 4-megapixel CCD camera (Scientific Instruments and Application, Duluth, GA, USA). Images were processed using Digital Micrograph Version 3.0 software (Gatan, Inc., Pleasanton, CA, USA).

### 2.6. Preparation and Visualization of Rho-HIS-Labeled “Spiraled Horn” Scaffolds

Horned scaffolds were prepared in 50 μL solution and washed with H_2_O as described above. The solid residue was then resuspended in 80 mM MOPS buffer, pH 7.1 (40 μL) and treated with NiCl_2_ (1.0 mM in DI H_2_O, 5.0 μL) for one hour. The solution was centrifuged at 10,000× *g* for three minutes at 4 °C. The supernatant (45 μL) was removed, and the solid residue was resuspended in 70 mM MOPS buffer, pH 7.1 (35 μL) and incubated with **Rho-HIS** (rhodamine-G_3_H_6_-NH_2_, 20 μM, 10 μL) for three hours. The solution was centrifuged at 10,000× *g* for three minutes at 4 °C, the supernatant (45 μL) was removed, and the solid residue was resuspended in DI H_2_O (45 μL). This process was repeated twice. The fluorescent scaffolds were placed in serum-free RPMI media (450 μL), plated in suspension onto glass slides, and visualized using a Nikon A1R-MP confocal microscope (Minato, Tokyo, Japan), equipped with a 561 nm (red) laser.

### 2.7. Cell-Binding Study

**Rho-HIS**-labeled horned scaffolds were prepared in 50 μL volume in a 1.5 mL Eppendorf tube as described above. HeLa cells were cultured in a T-75 flask with Dulbecco modified Eagle’s media (DMEM) complete media (10% Fetal Bovine Serum). Upon reaching 70%–80% confluency, the cells were trypsinized and treated with Roswell Park Memorial Institute (RPMI) media without added serum. Fifty-thousand HeLa cells were combined with the horned bundles and diluted with RPMI media to 500 μL total volume. Samples were mixed by inversion and incubated at 37 °C with 5% CO_2_ for two hours. Immediately prior to imaging, live cells were stained with Calcein AM (500 nM). Cells/bundle suspensions were plated onto glass slides and visualized using a Nikon A1R-MP confocal microscope, equipped with 488 nm (green) and 561 nm (red) lasers.

## 3. Results and Discussion

### 3.1. SEM Characterization of Assemblies

**Figure 3:**
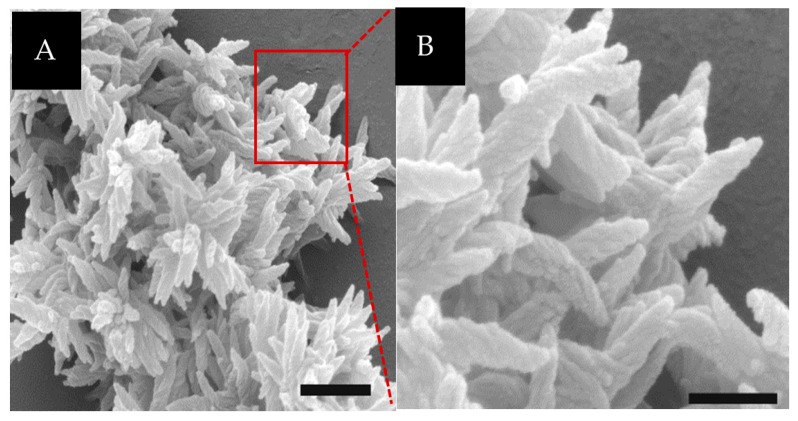
(**A**) SEM visualization of 1.0 mM **NCoH** and 1.0 mM **HisCol** with 2.0 mM CoCl_2_ (scale bar = 500 nm); (**B**) zoom-in of (**A**) (scale bar = 200 nm).

Incubation of 1.0 mM **NCoH** and 1.0 mM **HisCol** with 2.0 mM CoCl_2_ in buffered aqueous solution (pH 7.1) at room temperature for sixteen hours led to the formation of a dense white precipitate. After washing, this precipitate was visualized by scanning electron microscopy (SEM), revealing the presence of an intricate, three-dimensional matrix at the lower micron scale (Figure 3A). At first glance, the assembly appeared to resemble the petal-like network previously observed with 1:1 **HisCol**/**IdaCol** in the presence of Ni(II) [25]; however, closer examination revealed that, rather than smooth petals, these assemblies were composed of ruffled, spiraled components reminiscent of “unicorn horns” (Figure 3B). Each “horn” measured 300–600 nm in length and was found in distinct “bundles”, extending from central points. Interestingly, this “horned bundle” morphology appeared to be a unique combination of the petal-like network, previously observed with the **HisCol**/**IdaCol** system [25], with the ruffled surface morphology previously observed with **NCoH** microflorettes [24]. Curiously, incubation of this dual peptide system under identical conditions with ZnCl_2_, NiCl_2_, and CuCl_2_ failed to produce higher-order structures.

### 3.2. TEM Characterization of Assemblies

**Figure 4:**
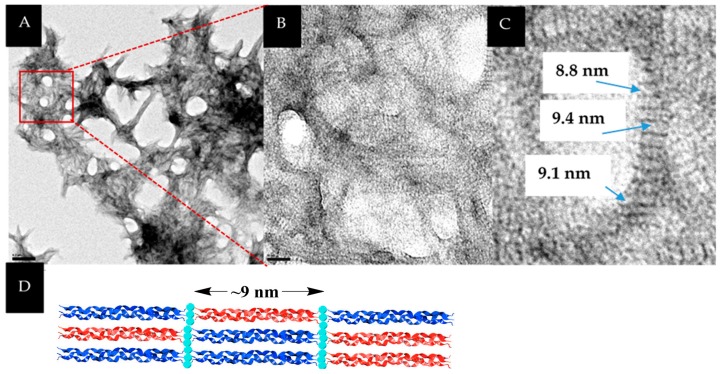
(**A**) TEM visualization of 1.0 mM **NCoH** and 1.0 mM **HisCol** with 2.0 mM CoCl_2_ (scale bar = 200 nm); (**B**) Close-up of (**A**) (scale bar = 20 nm); (**C**) close-up of (**B**) with bandwidth measurements; (**D**) cartoon of **NCoH/HisCol** assembly with the triple helix length.

Visualization of the **NCoH**/**HisCol** “spiraled horn” assemblies by transmission electron microscopy (TEM) revealed a highly interconnected network (Figure 4A). Closer examination of this network revealed periodic banding all throughout the assembly (Figure 4B) and within each horn. Moreover, the banding pattern (distance between the bands) was found to measure ~9 nm (Figure 4C). This distance corresponds to the length of a collagen peptide triple helix and could indicate the distinct placement of **NCoH** and **HisCol** peptide triple helices within the assembly (Figure 4D). Indeed, previous assemblies formed from a 1:1 mixture of **HisCol** and **IdaCol** were found to display a similar periodic banding pattern, presumably due to the tandemly-assembled repeats of the collagen peptide triple helices [25]. Likewise, periodic banding is observed in type-1 collagen fibrils, although with wider band gaps than our system due to our use of a more truncated triple helix [30,31].

### 3.3. The Effect of Strand Exchange within the Triple Helices

**Figure 5:**
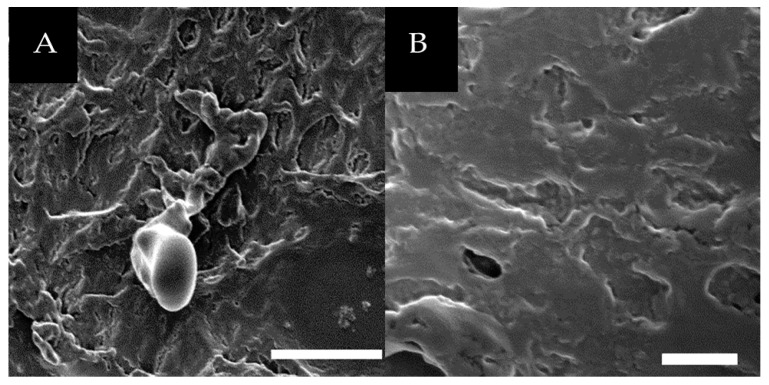
SEM visualization of (**A**) 1.0 mM **NCoH** and 1.0 mM **HisCol** thermally annealed prior to adding 2.0 mM CoCl_2_ (scale bar = 20 μm); (**B**) zoom-in of (**A**) (scale bar = 5 μm).

The proposed mechanism of assembly relies on the grouping of identical metal-binding ligands at each termini of the triple helices due to the parallel nature of collagen peptide triple helices. In this way, **NCoH** would not mix NTA and His ligands together at one terminus. To probe this mechanism, aqueous solutions of **NCoH** and **HisCol** were combined prior to addition of metal and subjected to thermal annealing by first heating to 90 °C, well above their respective melting points, for thirty minutes, in order to dissociate the triple helical units. The peptide solutions were then incubated overnight at 4 °C to allow the helices to reform. Reconstitution of the triple helices in this way may result in strand exchange between the peptide triple helices. Overnight incubation of these thermally-annealed peptides (1.0 mM of each) with 2.0 mM CoCl_2_ resulted in the formation of a thick, amorphous material with no distinguishing features (Figure 5), in no way reminiscent of the horned bundles formed by an analogous sample that was not subjected to thermal annealing (Figure 3A). These results serve to validate the importance of the positioning of the ligands within the triple helices for the formation of the intricate “spiraled horns” morphology. Similarly, the addition of the metal chelator EDTA to the horned structures served to remove all turbidity from the solution, verifying the importance of the availability of Co^2+^ in the assembly process.

### 3.4. Metal Concentration Dependency on Assembly Morphology

**Figure 6:**
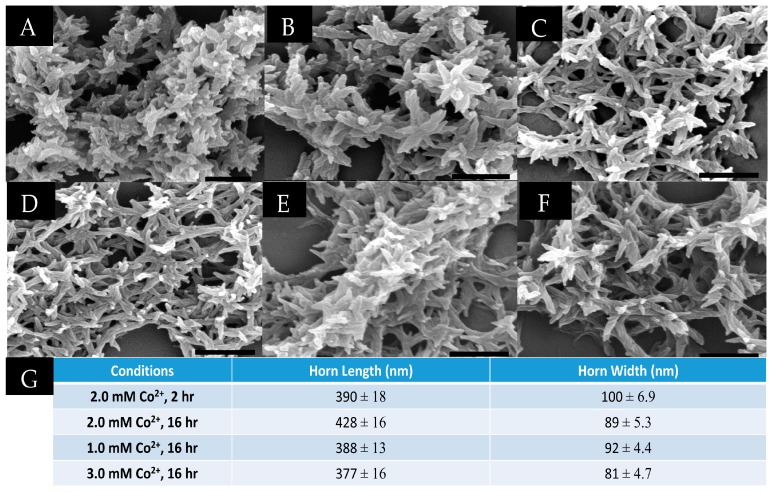
SEM visualization of 1.0 mM **NCoH** and 1.0 mM **HisCol** with (**A**) 0.5 mM CoCl_2_; (**B**) 1.0 mM CoCl_2_; (**C**) 3.0 mM CoCl_2_; and (**D**) 4.0 mM CoCl_2_ after overnight assembly (16 h); 1.0 mm **NCoH** and 1.0 mM **HisCol** with 2.0 mM CoCl_2_ (**E**) two minutes and (**F**) two hours after the addition of the metal (scale bars = 500 nm); and (**G**) average horn lengths and widths (nm) at the indicated conditions.

The mechanism of assembly was further tested by varying the concentration of Co^2+^ to which the collagen mimetic peptides were exposed. We had established that the bundles of ruffled horns, equivalent to a three-dimensional matrix, form after overnight incubation with 2.0 mM CoCl_2_. Interestingly, lowering the CoCl_2_ concentration to 0.5 mM and 1.0 mM resulted in no significant change to the established morphology, as, once again, significant levels of horned bundles were observed by SEM (Figure 6A,B). Increasing the Co^2+^ concentration to 3.0 mM and 4.0 mM produced a more loosely-packed morphology bearing closer resemblance to the petal-like networks previously observed with **HisCol**/**IdaCol** [25], however, spiraled horns remained visible throughout the network. (Figure 6C,D). Furthermore, the average horn lengths and widths of material produced under these conditions were found to be virtually identical (Figure 6G). Thus, it appears that the “horned bundle” morphology remains stable across a wide variety of metal concentrations, ranging from 0.5 to 2.0 mM. It was only when the metal concentration was raised to 3.0 mM and above that any real structural changes were noted, and, even then, such changes were fairly minimal.

### 3.5. Incubation Time Dependency on Assembly Morphology

Up to this point, all assembly studies were performed with an incubation time of sixteen hours. However, a cloudy precipitate was observed within the first thirty minutes after the addition of Co^2+^. Therefore, the material formed at earlier time points was examined to probe if precursor structures to the horned bundle morphology could be observed. To do so, samples were washed at the desired time points (to remove unbound metal ions), and immediately plated onto SEM stubs for visualization. Surprisingly, SEM micrographs taken of 1.0 mM **NCoH** and 1.0 mM **HisCol** with 2.0 mM CoCl_2_ revealed fully-formed horned bundles in as little as two minutes after metal addition (Figure 6E), which closely resembled the bundle morphology after overnight incubation (16 h) (Figure 3A). Visualization of an analogous sample after two hours of incubation with CoCl_2_ revealed that the resulting precipitate appeared very similar to the original sample, with a highly-intertwined network of well-formed horns (Figure 6F). Thus, Co^2+^-promoted self-assembly of **NCoH**/**HisCol** occurs very rapidly indeed, with a fully-formed, intricate morphology appearing within the first two minutes.

### 3.6. Decoration by a Histidine (His)-Tagged Fluorophore

**Figure 7:**
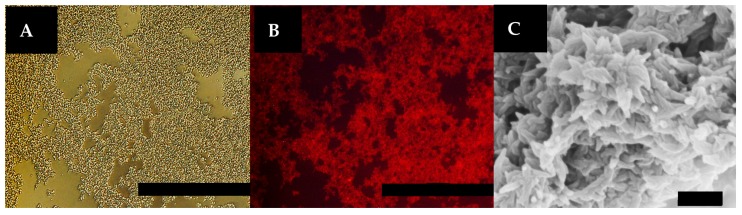
(**A**) Optical micrograph of **NCoH**/**HisCol** spiraled horn scaffolds, labeled with **Rho-HIS**; (**B**) red fluorescence micrograph of the same field of view (scale bar = 100 μm); and (**C**) SEM visualization of a **Rho-HIS**-labeled scaffold showing the spiraled horn morphology (scale bar = 500 nm).

Since the interactions between metal ions and ligands on the peptides were used to generate the assembly, it is likely that unsatisfied ligands exist within, and on, the surface of the horned bundles. Previous work performed with other metal-promoted CMP-based assemblies revealed the ability to decorate these assemblies using His-tagged biomolecules by exploiting these unsatisfied metal-ligand interactions [32]. Similarly, the **NCoH**/**HisCol** bundles should support functionalization by His-tagged molecules. Therefore, an attempt was made to functionalize the horned scaffolds with **Rho-HIS**, a rhodamine-based fluorophore containing a His tag (Figure 1C). To do this, the scaffolds were generated as described above with a 16 h incubation with CoCl_2_. The resulting precipitate was washed and treated with NiCl_2_ (1.0 mM in H_2_O, 5 μL) for one hour to enhance the affinity for surface-bound NTA ligands [32]. This was followed by incubation in the dark with **Rho-HIS** (20 μM in H_2_O, 10 μL) for three hours. After extensive washing, the material was visualized by fluorescence microscopy. A distinct red fluorescence was observed that was associated with the scaffold (Figure 7A,B). SEM visualization confirmed that addition of **Rho-HIS** has a negligible effect on the established “horn” morphology, as this intricate 3D matrix remained visible (Figure 7C).

### 3.7. Interaction between “Spiraled Horn” Scaffolds and HeLa Cells

**Figure 8:**
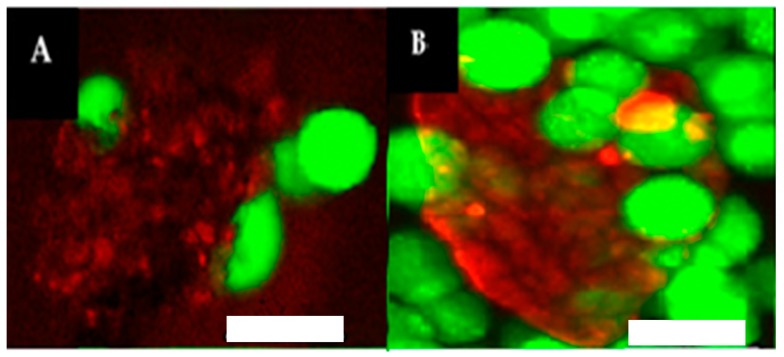
Fluorescent confocal visualization of spiraled horn scaffolds (labeled **red** with **Rho-HIS**) with live HeLa cells (stained **green** with Calcein AM) in media suspension (scale bar = 25 μm). (**A**,**B**) are two different areas of the matrix-cell complex.

The **NCoH**/**HisCol** horned scaffolds present an environment that is extended and multi-layered and that also has a ruffled surface, maximizing the available area for interaction. Moreover, unsatisfied metal-ligand interactions along the surface allow for decoration by His-tagged biomolecules, such as **Rho-HIS**. Furthermore, since collagen is abundant in the body, assemblies made from these collagen mimetic peptides should be biocompatible. With these attributes, the horned scaffolds may serve as a promising biomaterial for interactions with mammalian cells. A study was designed, therefore, using **Rho-HIS**-labeled horned bundles with HeLa cells suspended in cell culture media. HeLa, the immortal cervical cancer cell line extracted from the patient Henrietta Lacks in 1951 [33], was selected for this study due to the fact that it has proven itself to be remarkably robust and prolific, and, as such, is currently the most common cell lineage used for biopharmaceutical testing [34]. After a two-hour incubation at 37 °C with the cells and the collagen peptide bundles, live cells were stained with Calcein AM, and the samples were visualized by fluorescent confocal microscopy (Figure 8). The live, green HeLa cells were found associated with the surface of the red peptide scaffold. For instance, Figure 8A depicts two cells, one at the top left and one at the bottom right that are clearly embedded in the horned matrix. It is anticipated that increasing the concentration of the peptide matrix will result in more complete cellular encapsulation. Furthermore, it was previously observed with **HBN** that addition of live cells during scaffold assembly resulted in encapsulation [26]. It is highly likely that the present system will similarly facilitate encapsulation through cell addition during assembly. More work needs to be performed to more fully characterize the biological activity of the **NCoH**/**HisCol** horns. Nonetheless, these preliminary data indicate that the extended, ruffled surface of the horned bundles could be a satisfactory scaffold for cell interactions, suggesting further utility of the horned bundles as a cell-adhesive scaffold for regenerative medicine.

## 4. Conclusions

We have shown that the Co^2+^-promoted assembly of two collagen mimetic peptides, **NCoH** and **HisCol**, designed with metal-binding ligands at their termini, results in an intricate assembly, characterized by bundles of “spiraled horns”. These horned bundles displayed a periodic banding pattern all throughout the structure and within each horn, with banding lengths corresponding to the length of a single collagen triple helix, indicative of the assembly of distinct collagen peptide triple helices. Furthermore, strand exchange promoted by thermal annealing resulted in a dramatically different morphology with an amorphous assembly, suggesting the importance of ligand placement on the assembly process. Surprisingly, the horned bundle morphology proved to be remarkably resilient, forming as quickly as two minutes after metal addition and with little structural changes observed when the concentration of Co^2+^ was varied from 0.5 to 4.0 mM. Moreover, the horned scaffolds readily bound a His-tagged fluorophore and associated with live HeLa cells in suspension culture, providing preliminary evidence of biological functionality.
